# Effects of the Chinese Traditional Prescription Xiaoyaosan Decoction on Chronic Immobilization Stress-Induced Changes in Behavior and Ultrastructure in Rat Hippocampus

**DOI:** 10.1155/2013/984797

**Published:** 2013-12-02

**Authors:** Yuan Liang, Xiao-Ling Guo, Jia-Xu Chen, Guang-Xin Yue

**Affiliations:** ^1^School of Pre-Clinical Medicine, Beijing University of Chinese Medicine, Beijing 100029, China; ^2^Institute of Basic Theory in Chinese Medicine, China Academy of Chinese Medical Sciences, Beijing 100700, China; ^3^Fangzhuang Community Health Center, Beijing 100078, China; ^4^Department of Basic Theory in Chinese Medicine, Henan University of Traditional Chinese Medicine, Zhengzhou 450008, China

## Abstract

Xiaoyaosan (XYS) decoction has been widely used as a traditional medicine for treating stress and depression-related disorders in China for thousands of years. *Aim of the Study*. To observe the potential mechanism of XYS decoction's antidepressant-like effect in **α**-amino-3-hydroxy-5-methyl-4-isoxazolepropionic acid (AMPA) receptors related to synaptic plasticity in the hippocampus rats induced by chronic immobilization stress (CIS). *Materials and Methods*. Animals were randomly divided into five groups: (1) control group; (2) sham-operated group; (3) CIS group, in which rats were conducted CIS for 21 days; (4) XYS decoction treatment group; (5) 6-cyano-7-nitroquinoxaline-2,3-dione (CNQX) positive group, in which the amygdala of CIS rats was unilaterally microinjected with a competitive glutamate receptor antagonist, CNQX. After CIS for 21 days, the open field test (OPT) and elevated plus-maze test (EPM) were measured, the ultrastructure of hippocampus CA_1_ subregion was observed by the electron microscopy; both the GluR1 and GluR2 mRNA level of AMPA receptor subunits in hippocampus CA_1_ subregion were detected by real-time qPCR. *Results*. Rats subjected to CIS exhibited increases in time in central zone and decreases in total distance traveled in the OPT. In the EPM, they also showed decreases in center zone time and entries, open arm time and entries, and an increase in close arm time. Ultrastructural damage in the hippocampus CA_1_ was also observed. XYS decoction and CNQX showed significant improvement behavioral changes and the ultrastructural damage of the hippocampus CA_1_; XYS decoction also reversed CIS-induced decreases in GluR2 mRNA and increases in GluR1 mRNA in the hippocampus CA_1_ as well as CNQX. *Conclusions*. XYS decoction may effectively produce an antidepressant-like effect, which appears to be involved AMPA receptors related synaptic plasticity of hippocampus.

## 1. Introduction

There is abundant evidence demonstrating that chronic stress can cause hippocampal damage, such as dendritic remodeling, synaptic plasticity, dendrites retracting, decreased neurogenesis, and apoptosis [1]. Synaptic plasticity is one of the key factors indicating how stress response is transforming to damage; the changes of *α*-amino-3-hydroxy-5-methyl-4-isoxazolepropionic acid (AMPA) receptors in the synapses have a relation with synaptic plasticity [2]. However, stress induced anxiety and depression involve multiple areas of the brain, such as amygdale and the prefrontal cortex.

Both the hippocampus and the amygdala are important aspects of the limbic system which plays a dominant role in stress response. Anatomically, the amygdala projects to several hippocampal regions (including the CA_1_ area) [3, 4]. And functionally, both of them control the systems involved in stress. But it seems that they have different actions during stress response. There were findings about enlarged amygdala volume and reduced hippocampus volume in young women with major depression [5]. Considerable evidence indicates that the amygdala is critically involved in mediating stress-related effects on behavior and modulating hippocampal function. Furthermore, the hippocampus and anterior cingulate cortex inhibit stress-induced HPA activation, whereas the amygdala may enhance glucocorticoid secretion [6].

AMPA receptors mediate the majority of rapid excitatory synaptic transmission in the central nervous system [2, 7]. It is known that glutamate is a potent neuronal excitotoxin [8], and when it is combined with the AMPA receptor *α*-amino-3-hydroxy-5-methyl-4-isoxazolepropionic acid (AMPA) receptors, it produces depolarization and neuronal excitation. The excitation triggers either rapid or delayed neurotoxicity which may be an important cause of stress-induced neural damage. AMPA receptors are composed of a homo or heteromeric complex of four subunits, GluR1, GluR2, GluR3, and GluR4 [2]. In the mature hippocampus, most of the AMPARs are composed of GluR1-GluR2 or GluR2-GluR3 combinations [9]. AMPA receptors are mainly distributed in the postsynaptic membrane of the excitatory synapses. They are not static components of the synapses, but they are continuously being delivered and removed in and out of the synapses in response to neuronal activity [10]. The changes of the amount or certain subunits of the AMPA receptors may influence the activity and transmission effect of excitatory synapses. This dynamic process probably plays a key role in the synaptic plasticity that is thought to trigger aspects of learning and memory and also being one of the causative factors of neural damage under stress.

Our previous research [11] found that the expression of AMPA receptors decreased in hippocampus CA_1_ subregion while increased in basolateral nucleus of amygdala (BLA) after exposure to CIS, and XYS decoction had a regulation effect on the above changes.

Therefore, in the present study, 6-cyano-7-nitroquinoxaline-2,3-dione (CNQX), a competitive glutamate receptor antagonist, was microinjected in the amygdala to block the AMPA receptors as positive control. The purpose of this study was to further investigate the potential regulation mechanism of XYS decoction in synaptic plasticity relevant to the AMPA receptor in the central nervous system especially in hippocampus CA_1_ of rats with CIS.

## 2. Materials and Methods

### 2.1. Animals

A total of 100 male Sprague-Dawley rats (Beijing Weitong Lihua Research Center for Experimental Animals), weighing (230 ± 10) g, were used in the experiments. They were randomly divided into five groups: (1) control group; (2) sham-operated group; (3) CIS group; (4) XYS decoction treatment group (XYS group); (5) CNQX positive group.

The animals were housed in a room with routine care (20–24°C, relative humidity of 30–40%) and free access to food and water. This study was performed in strict accordance with the recommendations in the Guide for the Care and Use of Laboratory Animals of the P. R. China. The protocol was approved by the Committee on the Ethics of Animal Experiments of the Beijing University of Chinese Medicine (BUCM). All the surgery was performed under sodium pentobarbital anesthesia, and every effort was made to minimize animal suffering.

### 2.2. Preparation of Extracts of the XYS Decoction

The XYS decoction was composed of the following dried raw materials: Poria cocos (Schw.) Wolf (Poria), Paeonia lactiflora Pall. (Radix Paeoniae Alba), Glycyrrhiza uralensis Fisch. (Radix Glycyrrhizae), Bupleurum chinense DC. (Radix Bupleuri), Angelica sinensis (Oliv.) Diels (Radix Angelicae Sinensis), Atractylodes macrocephala Koidz. (Rhizoma Atractylodis Macrocephalae), Mentha haplocalyx Briq. (Herba Menthae), and Zingiber officinale Rosc. (Rhizoma Zingiberis Recens). These eight herbs were purchased from Medicinal Materials Company of Beijing Tongrentang and authenticated by Qi Junjie, BUCM, before processing the following procedures provided by the Department of Preparation for Herbs, Sino-Japan Friendship Hospital in Beijing, as previously described [12].

### 2.3. Surgery

All the rats except those in control group were anesthetized with pentobarbital sodium (40–50 mg/kg, i.p.) after adaption for three days. The animal was secured in a stereotaxic instrument by using nonpuncture ear bars and a bite bar. A stainless steel guide cannula (0.8 mm o.d.) was aimed to the right amygdala (coordinates: AP: −4.0 mm, L: 4.4 mm, DV: 8.0 mm) [13]. The cannula tip was 1.5 mm above the injection site, the cannula was attached to the skull bone with stainless steel screws and acrylic cement, and a stiletto inside the cannula prevented obstruction [14]. After the rats with CIS established for 1 week were performed surgeries, penicillin was given by the intramuscular injection to against infection. The histological site of cannula was verified by dye infusion at the end of the experiment, and demonstrated the accurateness.

### 2.4. Stress Procedure and XYS Treatment

The rats in CIS, XYS, and CNQX groups were carried out the immobilization stress 3 h per day for 21 days as described previously. The XYS decoction-treated rats were given via gavage the extracted mixture (5.32 g/kg) [15], 1 hour before CIS each day for 21 days. The rats in the other groups were given distilled water.

### 2.5. Intracerebral Injection

CNQX (Sigma-Aldrich Company), a selective AMPA receptor antagonist, was dissolved in 99.5% dimethyl sulfoxide (DMSO) (Sigma-Aldrich Company) and then diluted with sterile 0.9% saline to a final concentration of 0.5 *μ*g/0.5 *μ*L CNQX. Infusion was performed according to the method of Padovan et al. with minor modifications [14]. Briefly, a thin needle (0.4 o.d.) was introduced through the guide cannula until its tip was 1.5 mm below the cannula end. A polyethylene catheter (PE 10) was interposed between the upper end of the dental needle and the microsyringe. A volume of 0.5 mL was injected in 1 min using a Hamilton (USA) microsyringe. The movement of an air bubble inside the polyethylene catheter confirmed drug flow and the injector was left in place for an additional 1 min. Unilateral intra-amygdala infusion of CNQX (0.5 *μ*L) was administered to rats of CNQX group on the 1st, 4th, 7th, 10th, 13th, 16st, 19th, and 21st day; vehicle (sterile saline) was used as a control.

### 2.6. Behavioral Procedures: Open-Field Test (OFT)

The OFT was performed according to previously described procedure [12]. Briefly, at 8:00 of the 22nd day, all rats were put in a quiet, closed room around with black curtains for 10 min before the test, avoiding the unfavorable disturbances from the surroundings. Each rat was placed in the center zone (50 cm × 50 cm) of open-field box (100 cm × 100 cm × 40 cm) lightly; total distance traveled (cm) and time in central zone (sec) were recorded and calculated by EthoVision tracking software (Noldus Information Technology Inc., Holland). The apparatus was cleaned thoroughly with 95% ethanol after each animal was tested in case that the smell influenced the next rat.

*Elevated Plus-Maze Test (EPM).* The EPM test was performed the following procedures. After the OFT for two hours, each rat was put in the center zone of elevated plus maze with head faced to an open arm. Entry times in central zone, open arms and closed arms and time in central zone, open arms, and closed arms in 5 minutes were recorded and calculated by EthoVision tracking software. When one rat finished its test, the maze was cleaned by 95% ethanol.

### 2.7. Electron Microscopy

After the behavioral test, three animals of each group were analyzed by electron microscopy, and the animals were anaesthetized with 2% pentobarbital sodium (40 mg/kg) and perfused with cold 0.9% NaCl solution followed by perfusate solution (4% paraformaldehyde, 2.5% glutaraldehyde, and 0.1 mol PBS). Then, the brain was isolated and postfixed in 2.5% paraformaldehyde for 24 hours; the right CA_1_ region of hippocampus (2 mm × 2 mm × 2 mm) was cut out macroscopically from brain tissues.

For embedding electron microscopy, the sections were rinsed 8 times for 48 hours in 0.1 M phosphate buffer, postfixed with 2% osmium tetroxide for 1.5 hours, washed 5 times for 5 min in PBS, dehydrated through a series of alcohols acetone I (50%, 70%, 80%, 90%, 95%, and 100%, for 10 minutes each), further dehydrated in 100% acetone II, III twice for 40 minutes each, soaked in a ratio of 1 : 1 of solution of acetone and araldite resin for 1.5 hours, and then immersed in pure resin at room temperature for 3 hours, 37°C for 12 hours, 45°C for 12 hours, and 60°C for 48 h. Blocks were serially sectioned with a Leica UCT ultramicrotome into 70 nm. The sections were positioned onto 1 mm slotted, piloform-covered, copper coated grids, stained with 8% uranyl acetate and 0.4% lead citrate. Ultrathin sections were scanned at 80 kV with a JEOL-1230 transmission electron microscope (TEM).

### 2.8. Real-Time qPCR

Five samples of each group were detected by real-time qPCR. The animals were anaesthetized with 2% pentobarbital sodium (40 mg/kg) and decapitated and the brains were immediately removed on ice. Contralateral hippocampus CA_1_ region in individual animals was quickly isolated as Lein ES reported [16], put into 1.5 mL sterile centrifuge tube, and then frozen and stored at −70°C. Total RNA was extracted with Trizol reagent (Invitrogen) according to a standard protocol. The RNA was diluted in 40 *μ*L ultra pure water, and the RNA concentration was determined by NanoDrop ND-100 Spectrophotometer (Thermo Fisher Scientific, Wilmington, DE).

Total RNA from each sample was used to synthesize cDNA using High Capacity cDNA Reverse Transcription Kit with Gene Amp PCR System 9700 (Applied Biosystems, USA). A final concentration of 1 *μ*g/100 *μ*L was obtained. Real-time PCR primers were designed on the basis of the reported cDNA sequences. The sequences for primers are as follows: *β*-actin, forward 5′-CCTCTATGCCAACACAGTGC-3′ and reverse 5′-GTACTCCTGCTTGCTGATCC-3′; GluR1, forward 5′-GAGGGGAGGGAAGACCAA-3′ and reverse 5′-GCCGCATGTTCCTGTGAT-3′; GluR1, forward 5′-AGAGGACCCTTGTCTGTC-3′ and reverse 5′-GTGTTTGATGGCTTGAGT-3′; GluR2, forward 5′-CTACCAATGGGATAAGTTCGC-3′ and reverse 5′-TTCGCAGTCAAGGATTACACG-3′. Real-time PCR was performed using an ABI 7700 Real-Time PCR System (Applied Biosystems, USA) and an SYBR Green PCR Master Mix (Applied Biosystems) in a final volume of 25 *μ*L with the following thermal cycling conditions: 95°C for 30 min, followed by 40 cycles of 95°C for 15 s, 59°C for 20 s, 72°C for 20 s, and 83°C for 15 s. *β*-actin was used as an internal control.

Samples were assayed in triplicate. Relative amount of each expression of mRNA in each sample was calculated as the ratio of mRNA/*β*-actin. The relative amount of amplified product was performed using the comparative threshold cycle method as described in the manufacturer's manual.

### 2.9. Statistical Analysis

Data are expressed as means ± standard error of the mean (SEM). Differences among means were measured by one-way analysis of variance (ANOVA) and Student's *t* tests, the correlations corrected for multiple comparisons. Statistical significance was defined as *P* < 0.05.

## 3. Results

### 3.1. Behavioral Test

#### 3.1.1. OPT

**Figure 1:**
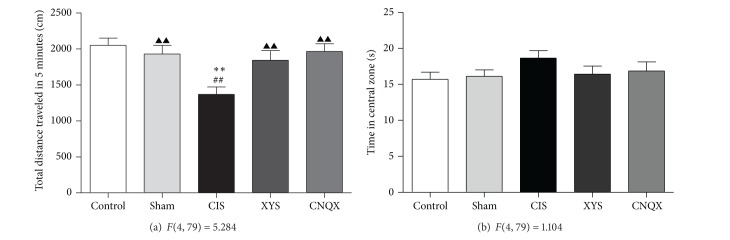
Performance of rats in the OPT: (a) total distance traveled in 5 minutes; (b) time in central zone of the test. Results are expressed as means ± SEM. **P < 0.01 versus control group, ^##^ *P* < 0.01 versus sham-operated group, and ^▲▲^ *P* < 0.01 versus CIS group.

The OPT is a behavioral test to measure the excited or depressed state of rats, commonly used to evaluate the exploratory behavior and reflect the emotional response of rats in new environment. In the present study, this test was used to verify whether the CIS model was successful. As shown in Figures 1(a) and 1(b), compared to the sham-operated rats, rats in CIS group showed decreased total distance traveled in 5 min (*P* < 0.01) and increased time in central zone (*P* > 0.05) after being put into the open field area. The decreased total distance traveled was effectively reversed by the treatment with CNQX or XYS decoction (*P* < 0.01); the time in central zone decreased after treatment, but no statistical difference was shown (*P* > 0.05). Both XYS and CNQX significantly ameliorated the behavioral abnormality of CIS rats.

#### 3.1.2. EPM

**Figure 2:**
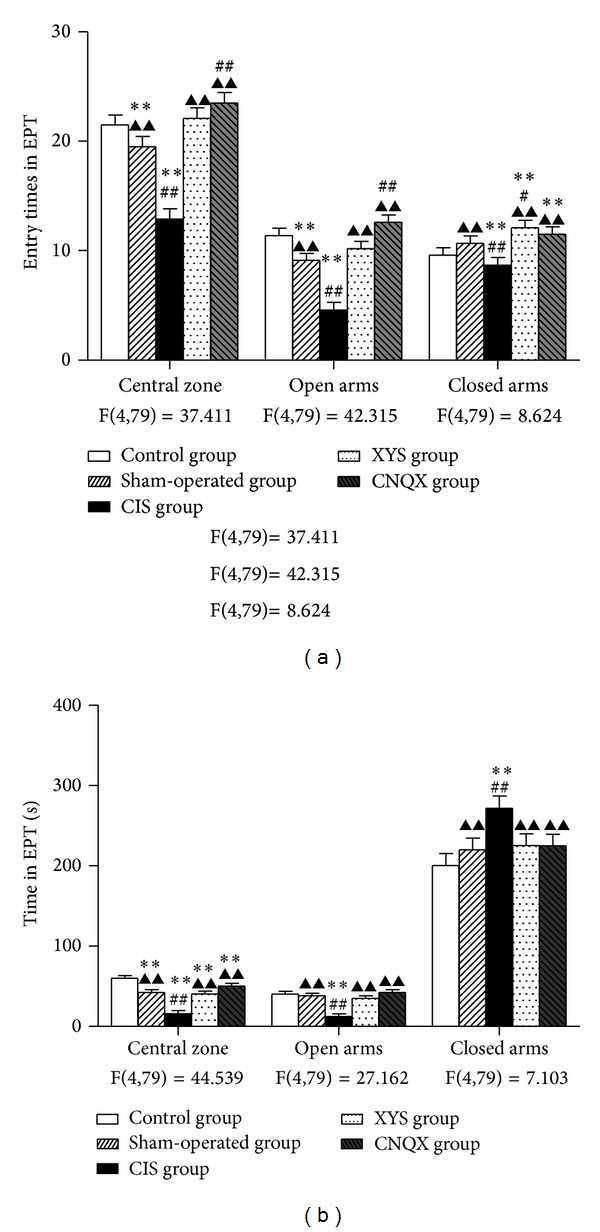
Performance of rats in the EPM: (a) entry times in central zone, open arms, and closed arms; (b) time spent in central zone, open arms, and closed arms in 5 minutes. Results are expressed as means ± SEM. **P < 0.01 versus control group, ^#^ *P* < 0.05  ^##^ *P* < 0.01 versus sham-operated group, and ^▲▲^ *P* < 0.01 versus CIS group.

The elevated plus-maze test is also commonly used as an anxiety test. CIS decreased the entry times and the time spent in central zone, compared to the control and sham-operated rats (*P* < 0.01, Figure 2(a); *P* < 0.01, Figure 2(b)). The decreases were reversed by the administration of CNQX (*P* < 0.01, Figures 2(a) and 2(b)) or XYS decoction (*P* < 0.01, Figures 2(a) and 2(b)).

Compared to the control and sham-operated group rats' activities, the entry times in open arms were decreased after CIS (*P* < 0.01, Figure 2(a)). Similar effects were found in the time spent in the open arms (*P* < 0.01, Figure 2(b)). XYS decoction increased the entry times and time spent in open arms (*P* < 0.01, Figures 2(a) and 2(b)). The CNQX showed better upregulation effects compared to the XYS and almost reached the normal level compared to the control group (Figures 2(a) and 2(b)).

No significant difference was found in the number of closed arm entries and time in the closed arms between control and sham-operated group (Figures 2(a) and 2(b)). The time spent in the closed arms was significantly increased after CIS compared to the control and sham-operated rats (*P* < 0.01, Figure 2(b)). XYS decoction decreased the time spent in closed arms; CNQX showed similar effects (*P* < 0.01, Figure 2(b)).

#### 3.1.3. TEM

**Figure 3:**
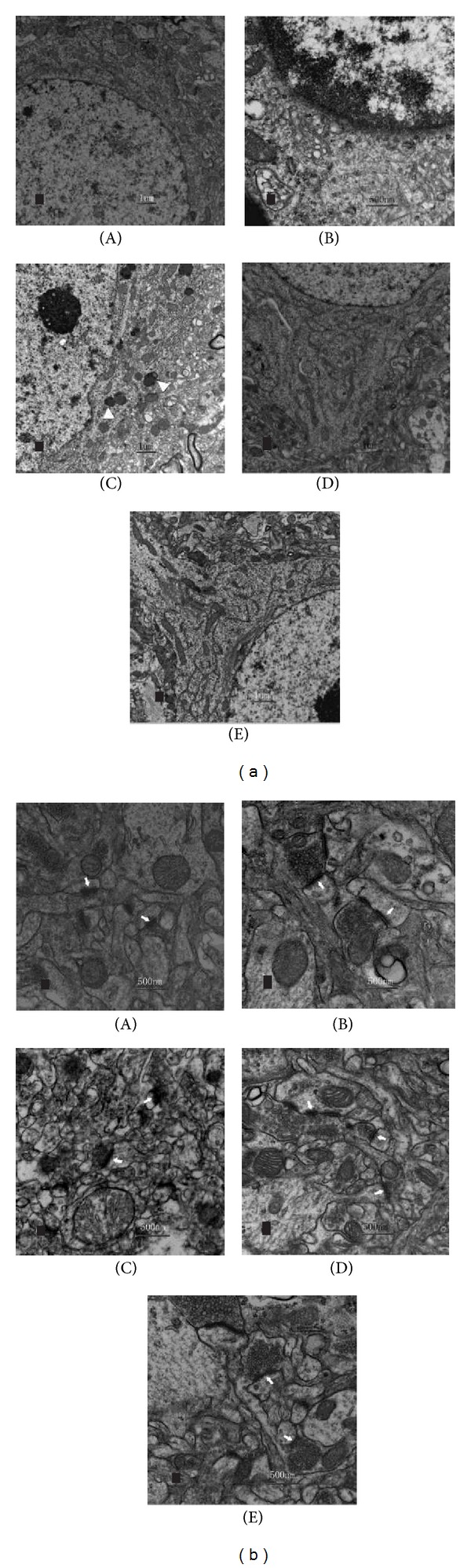
Ultrastructural changes of the hippocampus CA_1_ subregion in control (A), sham-operated (B), CIS (C), XYS (D), and CNQX (E) groups, respectively. (a) The morphology and structure of hippocampal pyramidal cells, including the nucleus and most of the organelles. Mitochondria, rough endoplasmic reticulum, Golgi's body, and free ribosome can be identified near the nucleus. A curly nucleus (C) and several cytolysosomes (triangle, (a)(C)) in the cytolymph are shown in CIS-induced hippocampus. (b) Synapses in hippocampus CA_1_. The synaptic structure is not clear and the fusion of pre- and postsynaptic membranes is visible in CIS-induced hippocampus (arrowheads, (b)(C)).

In subregion hippocampus CA_1_ of control and sham-operated control group, the membrane of hippocampal pyramidal cell nucleus was very smooth, and the caryotin was evenly distributed (Figure 3(a)). All kinds of organelles, morphologically normal mitochondria rough endoplasmic reticulum, Golgi's body, and free ribosome were identified easily near the nucleus (Figure 3(a)). The synaptic structure was normal; both pre- and postsynaptic membranes were clearly visualized; the synaptic cleft,spherical and vesicles were also clearly present (Figure 3(b)). The synaptic vesicles within these synapses were typically clear and round (Figure 3(b)). And of those in CIS group, nucleus of hippocampal pyramidal cell was shrunken and metamorphotic, exhibiting pyknotic nuclei, irregular, dispersed chromatin clumps, with deformation of mitochondria and several cytolysosomes in cytolymph (Figure 3(a)). Both the synaptic structure synaptic cleft and synaptic cleft were unclear, in contrast, the fusion of pre- and postsynaptic membranes could be visualized clearly (Figure 3(b)).

After XYS decoction treatment, no obvious changes were observed between hippocampus neurons and synapses compared with control group (Figures 3(a) and 3(b)).

#### 3.1.4. Real-Time qPCR

**Figure 4:**
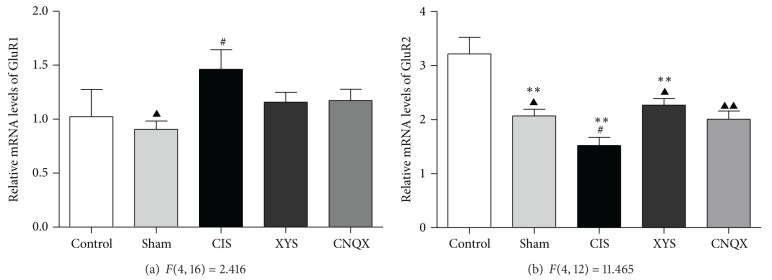
The expression of GluR1 and GluR2 mRNA in the hippocampus CA_1_. Results are expressed as means ± SEM. **P < 0.01 versus control group, ^#^ *P* < 0.05 versus sham-operated group, and ^▲^ *P* < 0.05  ^▲▲^ *P* < 0.01 versus CIS group.

Real-time qPCR analysis revealed that, in hippocampus CA_1_, 21-day stress increased the contents of GluR1 mRNA (*P* < 0.05, Figure 4(a)) in contrast to the sham-operated group; the level of GluR1 mRNA was decreased after XYS decoction treatment, but no significance was found comparing with CNQX positive control group (*P* > 0.05, Figure 4(a)). In contrast to the sham-operated group, the level of GluR2 mRNA was lower in the CIS group (*P* < 0.05, Figure 4(b)). However, XYS decoction treatment effectively reversed the GluR2 mRNA expression as well as CNQX positive group (*P* < 0.05, Figure 4(b)).

## 4. Discussion

OFT is one of the most popular assessment methods in behavioral research and often used to evaluate the environmental manipulations applying on the emotions of rodents [17, 18]. In our experiment, we additionally observed the animals' behavior by utilizing the EPM. This instrument has also been thoroughly validated as an extremely useful tool used to assess the emotional status of rodents [19]. Several variables can be measured in the open field; for instance, time spent in the center is considered to indicate emotional reactivity, and total distance traveled is thought to indicate locomotor and exploratory activity [17, 18]. The increased time spent in the center zone and decreased total distance of CIS rats showed a statement of limited spontaneous activity, anxiety, and depression, which caused decreased responsiveness, cognitive ability, and movement when the rats were placed in a novel environment. XYS decoction reversed CIS-induced changes, corroborating our previous findings [12], and CNQX showed the similar effect. This may reflect that both XYS and CNQX have some inhibitory influence on rat with chronic stress.

In our EPM test, 21 days' CIS rats showed increased occupancy of the closed arms and decreased occupancy of the open arms and center zone that are considered to indicate anxiety or depression. It is reported that animals with early restraint exhibited a significant decrease in the percent time spent and in the number of entries on the open arms; in addition, restraint induced a reduction in the total number of entries [20], which means exposure to hostile environment may produce behavioral and neuroendocrine changes that involve plasticity of central nervous system. In our investigation, it was found that XYS decoction effectively reversed CIS-induced behavior changes in EPM as well as CNQX positive control. These behavioral results of the present study clearly corroborated and extended previous findings that continuous administration of the XYS decoction exerts an antidepressant-like effect.

CNQX, a competitive AMPA (non-NMDA glutamate) receptor antagonist, was found to inhibit the locomotor activity of naive rats, and no symptoms of behavioral excitation were observed [21]. In our research, an increase in the exploratory behavior and motor activity in CIS rats after an intra-amygdala injection of CNQX was observed. These findings appear to provide that blocking AMPA receptors in particular brain regions might play an important role in relieving or aggravating depression-like behavior induced by chronic stress.

The results of electron microscopy showed that, in hippocampus CA_1_, a series of ultrastructural damages of cellular organs such as nucleus and mitochondria in pyramidal neurons and synaptic contacts were observed in CIS rats. Mitochondria are the main energy providers for the neurons. Mitochondrial damage or other dysfunction will cause serious obstruction in the production of ATP and reduction in the primary neural capacity and give rise to different changes in cells structure and function disorder. Information from one neuron flows to another neuron across a synapse. Decreased number or structure damage of synapses can induce obstruction of neuronal information transfer caused by changes of neurotransmitter delivery. Consistent with previous findings of repeated stress can cause loss of the pyramidal neurons [22], profound changes in the morphology of the mossy fiber terminals, and significant loss of synapses in both CA_3_ and CA_1_ of the hippocampus [23], we have proved that synaptic plasticity in the central nervous system was changed by chronic stress.

Chronic stress may serve as an experimental model to evaluate the underlying cellular and molecular alterations associated with some of the consequences of recurrent depressive illness. Our previous study found that, generally, CIS decreased the level of AMPA receptors in the hippocampus while increased them in the amygdala BLA [11]. The present data demonstrated that the level of GluR1 increased, whereas GluR2 decreased in the hippocampus CA_1_ after exposure to CIS. The opposite expression trends between GluR1 and GluR2 may be attributed to the differences in their process of synthesis, trafficking, and internalization. The high expression of GluR1 is probably attributed to the glutamate enhancement caused by chronic stress-induced HPA axis excitability, and the accumulated glutamate activates GluR1-containing AMPAR receptors [24, 25].

GluR2 expression may serve as a “molecular switch” leading to the formation of Ca^2+^-permeable AMPA receptors [26]. In fact, receptors containing the GluR2 subunit are calcium impermeable; only receptors lacking the GluR2 subunit (e.g., heteromers of GluR1/3) are calcium permeable [27, 28]. The position of GluR2 subunit in AMPA receptors can be replaced by GluR1 and GluR3 subunits when downregulation of GluR2 expression and stimulation of these calcium permeable receptors enhance excitotoxicity of endogenous glutamate following a neurological insult.

Some evidence has reported that mRNA and protein expression of the glutamate receptor subunit GluR2 in the hippocampus CA_1_ were suppressed by transient global ischemia, sustained or kainate seizures; CNQX altered the downregulation of GluR2 subunit and prevented the hippocampal neurons from the neurotoxicity [29–31]. During the chronic stress processed, the combined action of polygenic, multilevel, and multisignal pathways leads to the disorder of the immunologic functions of the hippocampus, hippocampal apoptosis, and proliferation disequilibrium [32]. This is consistent with the protective effect of microstructure of CNQX, and XYS decoction downregulation of GluR1 and upregulation of GluR2 in hippocampus CA_1_ respond to the 21-day stress in the present study.

It is well known that XYS decoction plays important roles in the clinical therapy of depression-related disorders in China [33], and several bioactive natural products have been identified from the herbs in the XYS decoction [33]. In Chinese medicinal theory, XYS decoction releases constraint and encourages the free-flow of Liver qi, allowing for open mindedness and rambling spirit [34]. Our previous clinic and experimental study demonstrated that the main ingredient of the XYS decoction is Radix Angelicas Sinensis (i.e., Chai Hu in Chinese), which acts as a nervous system sedative and a pituitary adrenocortical stimulant and is used for treatment of menstrual disorders [35–38].

In conclusion, this study demonstrated that XYS decoction can reverse the depression-like behavioral changes caused by CIS and produce an effect similar to the AMPA receptors in CNQX positive control, which indicates that one of the mechanisms of XYS decoction may attenuate the stimulation of the hippocampus CA_1_ to relieve its damage. Further research may be needed to prove that XYS decoction may effectively regulate the balance of the AMPA receptors excitability between hippocampus and amygdala.
